# Patient and caregiver perspectives on burden of disease manifestations in late-onset Tay-Sachs and Sandhoff diseases

**DOI:** 10.1186/s13023-020-01354-3

**Published:** 2020-04-15

**Authors:** Nicole Lyn, Ruth Pulikottil-Jacob, Camille Rochmann, Robert Krupnick, Chad Gwaltney, Nick Stephens, Julie Kissell, Gerald F. Cox, Tanya Fischer, Alaa Hamed

**Affiliations:** 1 Sanofi Genzyme, Cambridge, MA USA; 2 Sanofi Genzyme, Reading, Berkshire, UK; 3 IQVIA, Cambridge, MA USA; 4 Gwaltney Consulting, Westerly, RI USA; 5 Gerald Cox Rare Care Consulting, LLC, Needham, MA USA

**Keywords:** Late-onset GM2 gangliosidosis, Tay-Sachs disease, Sandhoff disease, Qualitative interviews, Disease manifestations, Burden of disease, Patient experience, Caregiver experience

## Abstract

**Background:**

The GM2 gangliosidoses (GM2), Tay-Sachs and Sandhoff diseases, are rare, autosomal recessive genetic disorders caused by mutations in the lysosomal enzyme β-hexosaminidase A *(HEXA)* or β-hexosaminidase B *(HEXB)* genes, respectively. A minority of patients have a late-onset form of disease that presents from late-childhood to adulthood and has a slowly progressive course with prolonged survival.

Little research has been published documenting patient experiences with late-onset Tay-Sachs and Sandhoff diseases and how the disease impacts their daily lives and functioning. This study explored the most frequent symptoms and functional impacts experienced by patients with late-onset GM2 gangliosidosis through interviews with patients and caregivers.

**Methods:**

A qualitative research study design was employed, using three focus groups and 18 one-on-one interviews with patients who were recruited at the National Tay-Sachs and Allied Diseases Annual Family Conference. Transcripts were generated from the discussions, and patient quotes were analyzed using a content analysis approach. Concepts were aggregated into symptom and functional impacts, and the frequency of mention in the focus groups and individual interviews was calculated.

**Key findings:**

Many of the frequently described symptoms [muscle weakness (*n* = 19, 95%), “clumsy” gait (*n* = 12, 60%), fatigue (*n* = 10, 50%)] and impacts [difficulty walking (n = 19, 95%), falling (*n* = 17, 85%), and climbing stairs (*n* = 16, 80%)] disclosed by patients and caregivers were similar to those previously reported in the literature. However, less frequently described symptoms such as gastrointestinal issues (*n* = 4, 20%) and coughing fits (*n* = 5, 25%) have been expanded upon. This study evaluated the immediate impact of these symptoms on the patients’ lives to highlight the burden of these symptoms and the functional limitations on daily living activities, independence, and emotional well-being. The findings were used to develop a conceptual disease model that could serve as a foundation for patient-centered outcomes in clinical trials and provide insights to the medical community that may benefit patient care.

**Conclusions:**

This study contributes to the current understanding of symptoms associated with late-onset GM2 gangliosidosis, and further identifies the many consequences and impacts of the disease. These symptoms and impacts could be measured in clinical trials to examine the effects of novel treatments from the patient perspective.

## Background

The GM2 gangliosidoses, Tay-Sachs disease and Sandhoff disease, are virtually clinically indistinguishable autosomal recessive lysosomal storage disorders caused by mutations in the genes encoding the α subunit (*HEXA*) or β subunit (*HEXB*), respectively, of the two β-hexosaminidase isoenzymes, hexosaminidase A (αβ) and hexosaminidase B (ββ). Tay-Sachs disease is caused by a deficiency of β-hexosaminidase A activity, whereas Sandhoff disease results from a combined deficiency of β-hexosaminidase A and B activities. The reduced ability of patients’ cells to catabolize GM2 ganglioside leads to progressive lysosomal accumulation of GM2 and related glycolipids, hence their classification as GM2 gangliosidoses [1]. Excessive accumulation of GM2 ganglioside occurs primarily within neurons, which leads to cell death [2] and progressive neurodegenerative symptoms.

Tay-Sachs disease is classified into three subtypes according to the clinical course: an acute infantile (classic) form with rapid progression and death before the age of 4 years, a subacute juvenile form with onset in early childhood and longer survival, and a chronic adult, or late-onset form (LOTS) with slower progression and longer survival [3]. Symptoms of late-onset Tay-Sachs disease include ataxia, dysarthria, muscle weakness, tremors, atrophy, and psychosis [4]. Muscle weakness predominantly affects the quadriceps and triceps muscles, which is a clue to the diagnosis. Other reported neurological presentations include spinal muscular atrophy, spinocerebellar ataxia and amyotrophic lateral sclerosis. Patients with late-onset Sandhoff disease manifest similar symptoms, including spinocerebellar ataxia, motor deterioration, sensorimotor neuropathy, tremor, dystonia, and psychosis [5]. The two diseases have much symptom overlap with few reported differences (e.g., greater prominence of speech difficulty in LOTS) such that they are viewed clinically as one entity. In late-onset GM2 gangliosidosis, symptoms typically develop in adolescence or early adulthood but can also appear later in life. The age and rate of progression on symptoms vary, but do not significantly affect longevity.

Very little peer-reviewed literature is available that documents the patient experience with late-onset GM2 gangliosidosis, and the existing literature comprises mostly case reports that describe the symptoms of individual patients or small groups of patients from a clinical, diagnostic, or natural history of disease standpoint. In a cohort of 21 patients, the authors reported that the majority of patients experienced balance problems and difficulty climbing stairs as well as prominent cerebellar signs of difficulty with verbal communication, writing, and eating. Due to muscle atrophy, most of the patients used assistive devices such as a cane or walker, or were wheelchair-bound (*n* = 6). Several patients experienced fine tremors of the outstretched hands, significant tricep muscle weakness, and leg weakness [4]. Similarly, the main symptoms in a cohort of 14 patients were articulation difficulties (*n* = 9, 64%), weakness of the lower extremities (n = 9), dysmetria, tremor, and gait instability (*n* = 8), and/or schizophrenic and maniodepressive disorders (*n* = 3) [6].

As the existing literature centers on the clinical presentation, particularly gait disturbances, balance problems and difficulty climbing stairs, there is limited data available on patient insights relating to their experience of living with the disease [4, 6]. This lack of data from patients and caregivers makes it difficult to understand the perceived burden and disease manifestations in late-onset GM2 gangliosidosis, particularly from the patient perspective. Qualitative methodology is well-suited to research that seeks to identify the common themes and varying perspectives among patients [7]. As it can be utilized with a smaller number of patients and facilitates the gathering of in-depth insight on the patient experience, it is highly applicable for rare diseases, including our population of interest [8]. The overall objective of this study was to explore and understand the symptoms of patients living with late-onset GM2 gangliosidosis and the impact of the disease on their lives.

## Results

### Participant demographics

Twenty-one unique patients with late-onset GM2 gangliosidosis participated in the study. Due to the poor audio quality of one interview, one individual interview could not be transcribed and therefore was excluded from analysis. In total, 20 transcripts were analyzed which included 17 individual interviews, two focus groups consisting of five (Patient Focus Group #1) or seven (Patient Focus Group #2) patients, and one caregiver focus group. Aggregated demographics of the 20 patients whose data were analyzed in the following sections are presented in Table 1. The seven caregivers who enrolled in the focus group interview in 2015 were typically close family members, including spouses or parents. The majority of caregivers were employed and living with the patient with late-onset GM2 gangliosidosis. All were aged 40 years or older and had been caregivers for more than a decade (Table 2).

**Table 1:** Demographics of patients participating in focus group and individual interviews

	Late-onset GM2 gangliosidosis patients (***n*** = 20)
**Mean age (range)**	49 (25–68)
**Male**	12 (60%)
**Female**	8 (40%)
**Late-onset Tay-Sachs disease**	16 (80%)
**Late-onset Sandhoff disease**	4 (20%)

**Table 2:** Demographics of caregiver focus group participants

	Late-onset GM2 gangliosidosis caregivers (n = 7)
**Age**	• 40–59 years: 43% • 60+ years: 57%
**Employment**	• Employed (full or part time): 71% • Retired: 29%
**GM2 disease variant of patient**	• Late-onset Tay-Sachs disease: 71% • Late-onset Sandhoff disease: 29%
**Current living situation**	• Living with and caring for spouse patient: 57% • Living with and caring for adult child patient: 29% • Living separately; visits sibling to assist with care: 14%
**Median time since beginning caregiving**	• 12 years (range 1–37 years)

### Saturation

Concept saturation was achieved for this study as limited new information emerged in the last group of the individual interview sample. Each group consisted of five interviews and were arranged in the order that the interviews were conducted. For symptoms, 90% of concepts emerged in the first group of interviews, followed by 5% in the second and third groups, and none in the last group. Seventy percent of impacts emerged in the first group of interviews, followed by 7% in the second group, 17% in the third group, and 5% in the fourth group. Four new impacts (jumping, sitting, internal body temperature, and stiffening) arose in the fourth group, but these concepts were deemed sufficiently similar to concepts elicited in earlier interviews.

### Signs and symptoms of late-onset GM2 gangliosidosis

In the 20 interviews, patients and caregivers discussed several key disease symptoms that impacted their lives. The most common symptom experienced by patients was muscle weakness, which was reported in 19 out of the 20 interviews (95%). Other common symptoms reported in interviews were “clumsy” gait (*n* = 12, 60%), fatigue (*n* = 10, 50%), and speech disorder (*n* = 9, 45%), specifically slurred speech or talking too quickly. Less common symptoms described by patients included muscle tremors, choking, coughing, swallowing, gastrointestinal issues, dry mouth, cramps, urinary retention, incontinence, hearing loss, itchiness, muscle tightness, numbness, pain, foot supination, swelling, tingling, memory loss, aphasia, and intellectual disability. Table 3 presents the signs and symptoms of late-onset GM2 gangliosidosis that were mentioned by 25% or more patients.

**Table 3:** Signs and Symptoms of GM2^a^

Concept	n (%)^b^
Muscle weakness	19 (95%) Spontaneous: 9 (45%) Probed: 10 (50%)
*Impact on driving*	*1 (5%)*
*Impact on getting dressed*	*1 (5%)*
*Impact on exercise*	*1 (5%)*
*Impact on recovery time*	*1 (5%)*
*Impact on needing assistance*	*2 (10%)*
*Worsened by fatigue*	*3 (15%)*
*Worsened by exercise*	*1 (5%)*
*Worsened by movement*	*1 (5%)*
“Clumsy” gait	12 (60%) Spontaneous: 4 (20%) Probed: 8 (40%)
Fatigue	10 (50%) Spontaneous: 8 (40%) Probed: 2 (10%)
*Worsened by walking*	*4 (20%)*
Slurred speech	9 (45%) Spontaneous: 5 (25%) Probed: 4 (20%)
*Worsened by conversation*	*2 (10%)*
*Worsened by dry mouth*	*1 (5%)*
*Worsened by stress*	*1 (5%)*
*Worsened by tiredness*	*1 (5%)*
Talking quickly	9 (45%) Spontaneous: 6 (30%) Probed 3 (15%)
*Worsened by stress*	*3 (15%)*
*Worsened by conversation*	*2 (10%)*
*Worsened by dry mouth*	*1 (5%)*
*Worsened by tiredness*	*1 (5%)*
Muscle tremors	6 (30%)
*Worsened by feeling nervous*	*2 (10%)*
Choking	5 (25%) Spontaneous: 4 (20%) Probed: 1 (5%)
Coughing	5 (25%)

^a^Signs and symptoms reported by less than 25% of patients included swallowing, gastrointestinal issues, cramps (n = 4, 20% each), dry mouth, memory loss (n = 3, 15% each), aphasia, urine retention, numbness (n = 2, 10% each), hearing loss, incontinence, intellectual disability, itchiness, muscle tightness, supination of the feet, swelling, and tingling (n = 1, 5% each)

^b^Number of patients who reported the concept out of a total of 20 interviews: 17 patient interviews, 2 patient focus group interviews, and 1 caregiver focus group interview. Concepts captured from focus group interviews were treated as a single interview during analysis as individual participants could not be differentiated in the focus group transcripts

Muscle weakness, particularly in the lower body, is the defining symptom of late-onset GM2 gangliosidosis and was mentioned in nearly every interview. One patient specified that “Quads, triceps, and core [are] the weak muscles for me” (Interview #9). Sometimes patients would use stronger muscles as support. One patient described needing to use his/her arms to get up from a seated position, saying “It’s more or less trying to push up with our hands. … to a standing position or I’d crawl over to something and pull myself up” (Interview #13). Patients often linked muscle weakness to falling and an inability to walk far distances because they believed their lower body muscles were too weak to sustain movement. One patient mentioned that “In my case, it’s if you even stumble just a little bit, your quads are not strong enough to stop you from falling” (Patient Focus Group #1). Similarly, another patient stated, “I’d say leg strength gets worse as I get fatigued. Like if I walk awhile, the chances that my legs are going to give out are greater” (Patient Focus Group #2).

A “clumsy” gait that made walking difficult and resulted in limited mobility was reported by 12 participants (60%) in the interviews. The term “clumsy” was used by many patients, and one patient stated that “… when you start falling, you’re just thinking you’re just clumsy. And I think that’s the first word that comes out of all of our mouths. … I feel I don’t pick my feet up as well because I’m scared of hitting that one pavement that’s off” (Interview #13). Clumsy gait was one of the first symptoms that patients noticed, and many had experienced this since childhood, even during important life events. One patient stated that “I just thought I was clumsy and I would fall down the stairs. I fell off the bleachers for graduation” (Patient Focus Group #2). Patients with late-onset GM2 gangliosidosis tended to adjust their stride to accommodate their difficulties in walking, and one patient mentioned that “If I bump into something, or something knocks me off-balance, I go right down. I fall quickly. So, when I walk and I move, I have to make sure that I’m secure, that I’m stable” (Interview #14).

After walking or climbing stairs, 10 patients (50%) reported feeling tired or fatigued and as a result would require resting for longer periods of time. One patient reported being more short-tempered due to fatigue, and another patient limited the duration of his/her daily activities. For one patient, the fatigue and difficulty climbing stairs limited the job opportunities that he/she was able to do, stating “It just wore me out to where I would come home and have no energy to do anything. … And I finally had to tell my boss that I just couldn’t do it anymore” (Interview #15).

Nine patients (45%) noticed a change in their speech as a result of their disease. This included slurred speech and/or talking too quickly, which had a direct impact on their ability to communicate verbally. A patient with slurred speech described it as speaking with “a heavy tongue and I also am beginning to kind of garble my words at times” (Interview #11). Patients with slurred or fast speech reported that their speech would worsen with stress, long conversations, dry mouth, or tiredness. Due to their speech difficulties, patients reported avoiding talking over the phone or they would reduce their conversations with others. To address these difficulties, five patients (25%) reported undergoing speech therapy, which helped one patient breathe and talk slower. None of the patients with late-onset Sandhoff disease experienced speech difficulties.

Muscle tremors were reported in six interviews (30%) and occurred primarily in the hands and legs. One patient who experienced muscle tremors in the legs described it as “If I’m sitting at a chair with my feet on the floor, I just…my feet sit on the floor and my knees will just shake” (Interview #16). Another patient mentioned that his/her hand tremors affected work, saying that “certain tools are hard for me to use. My hands shake. I used to be in the wine business, but pouring wine was difficult because my hands would vibrate, shake, and I would spill wine” (Interview #14). Patients reported that tremors affected their handwriting, stating “I’ve got severe hand tremors, but also my hands cramp extremely easily, so my handwriting is extremely messed up” (Interview #1).

Other recurring symptoms were long coughing fits (*n* = 5, 25%) that typically occurred when patients ate, as well as difficulties swallowing (*n* = 4, 20%). Choking without obvious triggers was mentioned by five patients (25%). One patient reported, “I have the choking as soon as I start eating, I’ll start coughing and it’s embarrassing because you’ll be at a restaurant and … People will ask you, are you okay and you can’t even answer them” (Patient Focus Group #1).

Four patients (20%) described experiencing gastrointestinal issues, including constipation and diarrhea. A patient reported one such incident, saying “I was out in a store and I was all of a sudden, within 10 seconds, I could feel it churning in my gut. I had to stop and could not move and I went right there. I had to run back to the bathroom because I couldn’t... It was twice and I had never… I hadn’t done that since I was six years old but twice within about three days I had to run home and change” (Patient Focus Group #1). Even though these symptoms were experienced some time ago, he stated that he will still only leave the house if the location has easily accessible toilets.

Issues with memory were reported in three interviews (15%), and ranged from short-term forgetfulness, such as “I get to the kitchen and I realize…why am I here? And it’s only been 20 seconds,” to long-term memory loss (Interview #16). One patient experienced aphasia, describing it as “from time to time I do lose words here and there. And I know the words but my brain … can’t access them sometimes” (Interview #11). A caregiver reported concerns with intellectual disability because the patient had difficulty with thinking rationally, describing the patient as “like a baby right now … There’s something wrong with his brain” (Caregiver Focus Group).

### Late-onset GM2 gangliosidosis-related impacts

Patients and caregivers experienced disease-related functional limitations centered on difficulties with gross motor function, balance, fine motor function, and other physical impacts. The physical manifestations of the disease affected patients in all aspects of their lives, and patients reported several emotional, social, work, and financial-related impacts. These impact domains are further explored in the following sections. Table 4 presents the disease-related impacts that were mentioned by 25% or more patients.

**Table 4:** Impacts of Late-onset GM2 gangliosidosis

Domain	Concept	n (%)^b^
Physical: 20 (100%)^a^	***Gross Motor Function: 19 (95%)***	
	Walking	19 (95%) Spontaneous: 8 (40%) Probed: 11 (55%)
	*Impact on activities*	*5 (25%)*
	*Impact on travel*	*4 (20%)*
	*Worsened by uneven surfaces*	*4 (20%)*
	*Worsened by hot/cold temperatures*	*2 (10%)*
	*Worsened by distance*	*2 (10%)*
	*Worsened by feeling nervous*	*1 (5%)*
	*Worsened by muscle weakness*	*1 (5%)*
	*Worsened by pain*	*1 (5%)*
	*Worsened by tremors*	*1 (5%)*
	Climbing stairs	16 (80%) Spontaneous: 8 (40%) Probed: 8 (40%)
	*Impact on leisure activities*	*3 (15%)*
	*Impact on vacation*	*1 (5%)*
	*Worsened by muscle weakness*	*2 (10%)*
	Getting up	16 (80%) Spontaneous: 11 (55%) Probed: 5 (25%)
	*Impact on driving*	*1 (5%)*
	*Needs assistance*	*4 (20%)*
	Gait	11 (55%) Spontaneous: 7 (35%) Probed: 4 (20%)
	*Worsened by speed*	*1 (5%)*
	*Worsened by uneven ground*	*1 (5%)*
	Lifting	7 (35%) Spontaneous: 5 (25%) Probed: 2 (10%)
	Knees buckling	6 (30%)
	***Fine Motor Function: 15 (75%)***	
	Dropping items	7 (35%) Spontaneous: 6 (30%) Probed: 1 (5%)
	Grip	6 (30%)
	*Impact on driving*	*1 (5%)*
	*Worsened by muscle weakness*	*1 (5%)*
	***Balance: 19 (95%)***	
	Falling	17 (85%) Spontaneous: 12 (60%) Probed: 5 (25%)
	*Impact on activities*	*4 (20%)*
	*Impact on getting dressed*	*1 (5%)*
	*Impact on showering*	*1 (5%)*
	*Worsened by tiredness*	*3 (15%)*
	*Worsened by speed*	*2 (10%)*
	*Worsened by not concentrating*	*2 (10%)*
	*Worsened by exertion*	*1 (5%)*
	*Worsened by illness*	*1 (5%)*
	*Worsened by walker*	*1 (5%)*
	Broken bones	12 (60%) Spontaneous: 9 (45%) Probed: 3 (15%)
	*Resulting in surgery*	*5 (25%)*
	*Impact on sleep*	*1 (5%)*
	*Worsened by falling*	*3 (15%)*
	Standing	5 (25%) Spontaneous: 4 (20%) Probed: 1 (5%)
	***Speech: 5 (25%)***	
	Speech	5 (25%) Spontaneous: 3 (15%) Probed: 2 (10%)
	*Worsened by tiredness*	2 (10%)
	*Worsened by stress*	2 (10%)
	*Worsened by cold*	1 (5%)
Emotional: 18 (90%)^b^	Fear of falling	10 (50%) Spontaneous: 6 (30%) Probed: 4 (20%)
	Frustrated	9 (45%) Spontaneous: 8 (40%) Probed: 1 (5%)
	Embarrassed	6 (30%)
	*Impact on social activities*	*1 (5%)*
	Scared/worried	6 (30%)
	*Impact on daily activities*	*3 (15%)*
	Self-esteem	5 (25%) Spontaneous: 4 (20%) Probed: 1 (5%)
	Cautious	5 (25%)
Financial: 13 (65%)^c^	Inability to work	7 (35%) Spontaneous: 5 (25%) Probed: 2 (10%)
Social: 18 (90%)^d^	Difficulty communicating	11 (55%) Spontaneous: 8 (40%) Probed: 3 (15%)
	*Impact on ordering at restaurants*	*1 (5%)*
	*Worsened by talking quickly*	*1 (5%)*
	Social activities	8 (40%)
	Negative impact on relationships	7 (35%)
	*Impact on relationship with friends*	*4 (20%)*
	*Impact on relationship with children*	*2 (10%)*
	*Impact on relationship with partner*	*2 (10%)*
	*Impact on relationship with family*	*1 (5%)*
	*Worsened by speech*	*1 (5%)*
Work: 18 (90%)^e^	On disability insurance	10 (50%)
	Productivity	6 (30%) Spontaneous: 5 (25%) Probed: 1 (5%)
	Reduced work hours	5 (25%)

^a^Physical impacts reported by less than 25% of patients included sprain, pain, sports/exercise (n = 4, 20%), bruising, carrying items, picking up items, sleeping (*n* = 3, 15%), typing, writing, slow reaction time, stutter (*n* = 2, 10%), jumping, sitting, throwing, chewing, abscesses, eyesight, internal body temperature, longer time to heal, judging distance, soreness, stiffening (*n* = 1, 5% each),

^b^Emotional impacts reported by less than 25% of patients included sad/depressed (*n* = 4, 20%), anxiety (*n* = 3, 15%), anger (*n* = 2, 10%), feeling drained, short tempered, and stress (*n* = 1, 5% each)

^c^Financial impacts reported by less than 25% of patients included medical bills (*n* = 4, 20%), purchasing specialty items, job limitations (*n* = 3, 15% each), moving to more accommodating residence, dependent on others (*n* = 2, 10% each), medical complications, specialty adaptation to home, and travel (*n* = 1, 5% each)

^d^Social impacts reported by less than 25% of patients included social withdrawal, dependent on someone (n = 4, 20% each), difficulty with romantic relationships (n = 3, 15%), difficulty making friends, treated differently, and talking less (n = 2, 10% each)

^e^Work impacts reported by less than 25% of patients included changed jobs (n = 4, 20%), limited job opportunities, unemployed, retired, fired (n = 3, 15%), difficulty finding job (n = 2, 10%), lack of progression, and quit (n = 1, 5%)

#### Functional limitation: gross motor function (*n* = 19, 95%)

Many of the difficulties with gross motor function stem from the muscle weakness that predominantly affects patients with GM2 gangliosidosis. In nearly every interview, patients and caregivers mentioned experiencing limitations associated with gross motor function. Patients’ gross motor function was sometimes further worsened by disease-related symptoms, such as muscle weakness or tremors, or pain from prior falls. Generally, patients perceived their limited mobility (*n* = 19, 95%) to be one of the greatest direct consequences of the disease, as it results in an impact on their daily activities, particularly in regards to their inability to walk without assistance, get up from a seated position or after falling, or go up a flight of stairs. One patient described the need to plan different outings, such as “if I have to go grocery shopping or something, I take my walker, but I have to strategize every move I make as far as bringing the cart to my car” (Interview #10). Difficulty getting up from a seated position was linked to muscle weakness and one patient noted “I need something to lean on to get just leverage … because I don’t have enough strength in the quads to just stand up” (Patient Focus Group #1). Being unable to climb stairs was particularly frustrating for patients as it affected their choice of leisure activities, jobs, and housing.

Many patients used adaptive devices: 12 patients (60%) used walkers, 11 patients (55%) used wheelchairs, seven patients (35%) used canes, three patients each (15%) used braces, chairlifts, or other forms of support (e.g., handrail), and two patients (10%) used a scooter. Eleven patients (55%) reported that they adapted their gait, either to help them walk for longer or prevent falling while walking. This adapted gait was described as “I have to lock my legs … I don’t really just kind of walk as a stride. I kind of almost like shift my weight back and forth … my quads are so atrophied that … the only way I can really walk is to lock my legs straight” (Interview #16).

#### Functional limitation: balance (*n* = 19, 95%)

As a consequence of poor balance, patients fall frequently, which leads to traumatic memories and a constant fear of falling for patients who were still mobile. Any small missteps or uneven surfaces could trigger a fall. Walking and falling typically were mentioned together, and one patient noted that “You get frustrated because you’re scared to walk. I notice instead of watching really where you’re going, you’re constantly looking down at where you’re walking” (Interview #13). Due to the limited mobility and increased risk of falling, the ability of these patients to perform their daily activities is severely reduced. Daily tasks such as fixing one’s hair could result in severe consequences, such as one patient who was “doing my hair in the bathroom and I got too weak and I fell backwards and I hit my head on the railings in the tub” (Patient Focus Group #2). After falling, some patients who were unable to get up on their own were left to wait for assistance. One caregiver noted that “if she cannot get up, she will be sitting there, on the floor, waiting for me. Then I’ll get the phone call, “Mom, where are you? Can you come fast? I want to go to the bathroom,” because… She’s left alone” (Caregiver Focus Group). A variety of emotions were associated with patients’ balance and falling. One patient was still embarrassed by an incident that occurred many years ago, in which he “was walking down some stairs, walking with the principal of the school, and I lost my balance and this 500-sheet stack of paper started going down” (Patient Focus Group #1).

#### Functional limitation: fine motor function (*n* = 15, 75%)

Patients reported trouble with fine motor function which included an impact on their ability to maintain a grip (*n* = 6, 30%), drop items (*n* = 7, 35%) as well as difficulty carrying and picking up items (*n* = 3, 15% each). Some examples of difficulty with fine motor function included “throwing something, tossing something up, or closing the car door I notice that it’s very difficult for me unless I really focus on it because it’s weaker” (Interview #10), with many patients stating that these difficulties stem from muscle weakness and hand coordination. This difficulty with fine motor function impacts patients’ independence, as they have to depend on others to drive them, open cans or bottles, or cut food. One patient stated that for “steak or chicken, I’ll just let somebody cut it for me. Because of strength, not clumsiness” (Interview #9). Caregivers often would help patients with tasks that require fine motor function, and one caregiver stated, “You have to cut a piece of meat because they cannot do it. You have to pick up something from the floor because it always goes down” (Caregiver Focus Group).

#### Functional limitation: physical (*n* = 13, 65%)

Patients and caregivers reported a number of additional physical impacts, including pain, effect on sports/exercise (*n* = 4, 20% each), and sleeping (*n* = 3, 15%). Four patients (20%) reported pain in the knee or unspecified locations and noted that it worsened with certain actions such as walking or laying down. Due to previous falls, one patient experienced knee pain and had to undergo surgery to repair broken shoulders and knees. Another patient also commented on the effect of broken bones, as broken ribs affected his/her ability to sleep peacefully, and due to the disease “it takes longer to heal or longer to get things done” (Interview #14). A caregiver stated that the pain limited the patient’s activities, as “he used to do a lot of things that he won’t do now because of the pain. So it just limits him a little bit more” (Interview #6). Due to muscle weakness, one patient reported limitations in his/her exercise, stating “I used to ride a bike longer distances than I can now … I don’t go as fast as I used to” (Interview #8).

#### Functional limitation: speech (*n* = 6, 30%)

Due to patients’ slurred speech and talking too quickly, six patients (30%) reported impacts of their speech difficulties which included unspecified speech impacts (i.e., patients did not expand further on their speech difficulties) (*n* = 5, 25%) and stuttering (*n* = 2, 10%). A patient described his/her speech as both slurred and fast paced, specifying that “I’ve been told by other people … my speech is still understandable, it’s just sometimes I slur things. And I think that happens because I speak fast without thinking about it” (Interview #4). One caregiver in attendance during the patient interview noted that speech was the most bothersome symptom because “if they can’t understand her and she can’t get her thoughts out clearly, then that’s her biggest issue” (Interview #8). Another patient noticed that he/she stuttered occasionally due to dry mouth, stating “In an interview, my mouth gets dry so I stutter a lot more” (Interview #1).

#### Emotional (*n* = 18, 90%)

Fear, frustration, embarrassment, and worry were the primary emotions of focus in the interviews. In half of the interviews, patients and caregivers reported a fear of falling while walking or getting up from a seated position, and many patients had experienced multiple broken bones and fractures from falling. Even patients who did not have a significant difficulty with immobility still had a constant fear of falling. Since there is always the concern that one might fall, a patient noted that “every time I do anything, taking steps or doing anything, I’m always very, very vigilant and just being very careful” (Interview #16). Constant falls led to an overall feeling of frustration as one patient noted, “I get aggravated, and I start screaming. It’s embarrassing as all heck, but I scream” (Patient Focus Group #2).

Patients often felt frustrated due to their speech and muscle weakness. Since patients had slurred speech or spoke too quickly, it was difficult to communicate with others and negatively impacted some patients’ social lives. A caregiver noted that the patient he/she cared for often felt frustrated since it was difficult for others to understand the patient, who “will always come home and say [unintelligible] and just kind of like give up. Some people just say ‘Huh? Huh?’ … He’ll order a lager and they give him a water and he’ll get annoyed” (Interview #6; from caregiver in attendance).

Embarrassment was centered on symptoms, such as muscle weakness, gastrointestinal issues, and coughing, as well as impacts such as falling and going out. One patient was embarrassed by how muscle weakness prevented him/her from being able to stand up to use the bathroom, stating “that’s really bad for my psyche and ego … I wear “Depends” and I have my help clean it up at night” (Interview #4). One patient mentioned that the frequent coughing was embarrassing as it caused a lot of unwanted concern from others, particularly when “going out to eat and then you eat and you’re either…you start that cough and people think you’re constantly choking” (Interview #13).

A number of patients were worried about disease progression and worsening symptoms. After a bad fall that resulted in a broken patella, one patient was worried, stating “I was scared I would never walk again” (Interview #14). Though some patients were worried about falling, another patient specified that his/her fear was not of falling, but of not being able to get up again. Lastly, the fear of the future and how quickly the disease might progress was a tangible concern, and a patient stated that “I’ve noticed over the last probably six months that I’m definitely starting to progress…not quickly, but much quicker than I have in the previous four to five years” (Interview #16). What was even more concerning for this patient was that it would be many years before he/she would be able to collect a disability check, and “I don’t know if I’m going to be able to make it that far” (Interview #16).

#### Social (*n* = 18, 90%)

Patients were acutely aware of the effect that late-onset GM2 gangliosidosis has on other people. They felt that their disease is burdensome to others and that these feelings made it difficult for them to proactively engage with other people. Difficulty communicating was reported in 11 interviews (55%) and affected communication with family, friends, and strangers. Despite patients’ best efforts to communicate, “some people won’t take the time to try and listen” (Interview #6).

Due to the actual and perceived social hurdles, patients and caregivers felt that their disease negatively impacted relationships as “friends, family, everything, his whole life was affected by this” (Interview #6). Engaging in romantic relationships was difficult, particularly for one patient who “used to be engaged and it never worked out because she couldn’t handle my condition” (Interview #4). Another patient mentioned that having the disease was very challenging for his/her self-esteem to handle having “such a difficult diagnosis, looking for a partner. Most women, they’ll run for the hills when they see that” (Interview #11). One patient felt socially isolated due to limited mobility, stating “I can’t dance at weddings. When people go to weddings and they are getting up to do the Hora or whatever, I am not and I am not too happy about that. My girl got married two years ago and I was sitting down. My husband danced, but I could not dance” (Patient Focus Group #1).

#### Work (*n* = 18, 90%)

Most patients were unable to work in the career of their choice due to the physical limitations of the job, communication obstacles, or financial constraints. Six patients (30%) reported that the disease affected their productivity, as muscle weakness affected jobs that ranged from physical labor –“I just could not handle the climbing up and down in the truck all day long” (Interview #15) to fine motor skills – “He had problems with the weakness and typing in the computer” (Interview #14). A caregiver stated that due to the patient’s worsening speech difficulties, “she ultimately lost her job. … So rather than having a career in marketing, she’s a cashier at a supermarket. …. Her career is no longer an option for her” (Interview #8). Lastly, patients had few opportunities available as the job needed to meet certain requirements, such as “I have to get a job that I could do in my wheelchair. And I have to get a job that’s close enough to home for me … That dictates where I can work” (Interview #1).

#### Financial (*n* = 13, 65%)

Patients and caregivers were significantly impacted financially due to disease management or securing their future financial stability. Patients expressed an overwhelming concern about being eligible for or staying on disability (*n* = 10, 50%). Being unable to work caused financial difficulties and many patients needed to live with their parents for symptom and financial support. As one patient was unable to work a full-time salaried job and was on disability, her caregiver stated that “The disability only allows her to make a certain amount of money a month, which limits whatever kind of job she can get. … She could no longer afford to support herself” (Interview #8). Due to their disease symptoms, patients often had to purchase specialty items, such as an electric chair to navigate stairs, and others made changes to their homes such as “put[ting] a foam floor on the top of a packed … floor, so when she falls, it’s not as bad” (Caregiver Focus Group). When a patient mentioned in a focus group that “I will have to sell my house and move to a one level eventually” (Patient Focus Group #1), others also echoed the need to move to a more accommodating residence. Since some patients required non-medical services, such as a trainer or nursing service, these were not covered by health insurance and required out-of-pocket payment. Patients were acutely aware of the amount of money they earned in order to remain eligible for Medicaid, which was necessary as “prescriptions are over $1,000 a month, so I can’t be without Medicaid” (Interview #1).

### Overall perspectives on late-onset GM2 Gangliosidosis

Patients were asked to consider what the most bothersome symptoms of late-onset GM2 gangliosidosis were and which were the most important to treat. Due to time constraints, not all patients were asked these questions. Five patients felt that the most bothersome symptom was muscle weakness, followed by lack of independence (*n* = 3) and speech and walking (*n* = 2 each). For treatment, four patients each felt that preventing disease progression and being able to walk would be the most important treatment benefits. A meaningful improvement in symptoms would be reducing muscle weakness (*n* = 4), improving the ability to communicate, improving clarity of speech, and not needing to use a wheelchair (n = 2 each). Overall perspectives are presented in Tables 5 and 6.

**Table 5:** Late-onset GM2 gangliosidosis-related adaptations

Concept	n (%)^b^
Walker	12 (60%)
Wheelchair	11 (55%)
Adaptation to home	8 (40%)
Cane	7 (35%)
Physical therapy	6 (30%)
Hyperextension of legs	3 (15%)
Speech therapy	5 (25%)
Service dog	4 (20%)
Changed car	3 (15%)
Brace	3 (15%)
Chairlift	3 (15%)
Other walking support	3 (15%)
Aqua therapy	2 (10%)
Scooter	2 (10%)
Swallowing therapy	1 (5%)
Occupational therapy	1 (5%)
Therapist	1 (5%)

**Table 6:** Most bothersome, meaningful improvement, and important to treat concepts

Concept	n^a^
Most Bothersome	
Muscle weakness	5
Lack of independence	3
Speech	2
Walking	2
Anxiety	1
Balance	1
Coughing	1
Difficulty communicating	1
Disease progression	1
Falling	1
Gastrointestinal issues	1
Getting up	1
Inability to climb stairs	1
Pain	1
Self esteem	1
Social life	1
Meaningful Improvement	
Reduce muscle weakness	4
Improve ability to communicate	2
Improved clarity of speech	2
No wheelchair	2
Keeping pace with others	1
Reduce difficulty speaking	1
No falls	1
Ability to climb stairs	1
Walk further	1
Walk without assistance	1
Most Important Change: 1st	
Walking	4
Speech	3
Getting up	3
Falling	2
Balance	1
Mobility	1
Most Important Change: 2nd	
Muscle weakness	2
Speech	2
Coughing	1
Gastrointestinal issues	1
Internal body temperature	1
Walking	1
Most Important Change: 3rd	
Falling	1
Controlling breathing while speaking	1
Insomnia	1
Most Important to Treat	
Prevent disease progression	4
Walking	4
Getting up	3
Measurable improvement	3
Speech	3
Muscle weakness	2
50% fewer bathroom trips	1
50% less coughing	1
Balance	1
Fatigue	1
Without side effects	1

^a^Not all patients were asked or reported their most bothersome, important to treat, or meaningful improvement. Thus, only the number of patients is reported

### Caregiver perspectives on patients’ experiences

Caregivers provided valuable insight into how the disease affected the patients they cared for. They felt that patients struggled to accept their condition, were anxious about the future, and experienced emotional isolation, particularly those with moderate to advanced disease. Additionally, caregivers noted that patients are acutely aware of their limited capabilities and their symptom progression over time (patients in individual interviews similarly described how their disease has progressed). Caregivers noted that many patients experienced frustration in their inability to self-care, help their caregivers around the household, and move independently. One caregiver stated that, “I think the biggest problem [he] had was stopping driving. That was his last vestige of independence. It didn’t matter that he was in a wheelchair” (Caregiver Focus Group). With the progression of disease over time, caregivers expressed that patients felt increasingly isolated as a result of their reduced ability to express themselves or actively engage with others in social situations.

The aspects of late-onset GM2 gangliosidosis perceived by caregivers to be most burdensome for patients were the lack of independence, impact on self-esteem, and muscle weakness, confirming patient reports that muscle weakness was among their most bothersome symptoms. Caregivers noted that the lack of independence and impact on self-esteem were often linked. For one caregiver, the patient who he/she cared for “feels he is a burden. It doesn’t matter what I say or do. I can’t seem to help him overcome that lacking that he has” (Caregiver Focus Group).

### Conceptual model of late-onset GM2 Gangliosidosis symptoms and functional limitations

**Fig. 1:**
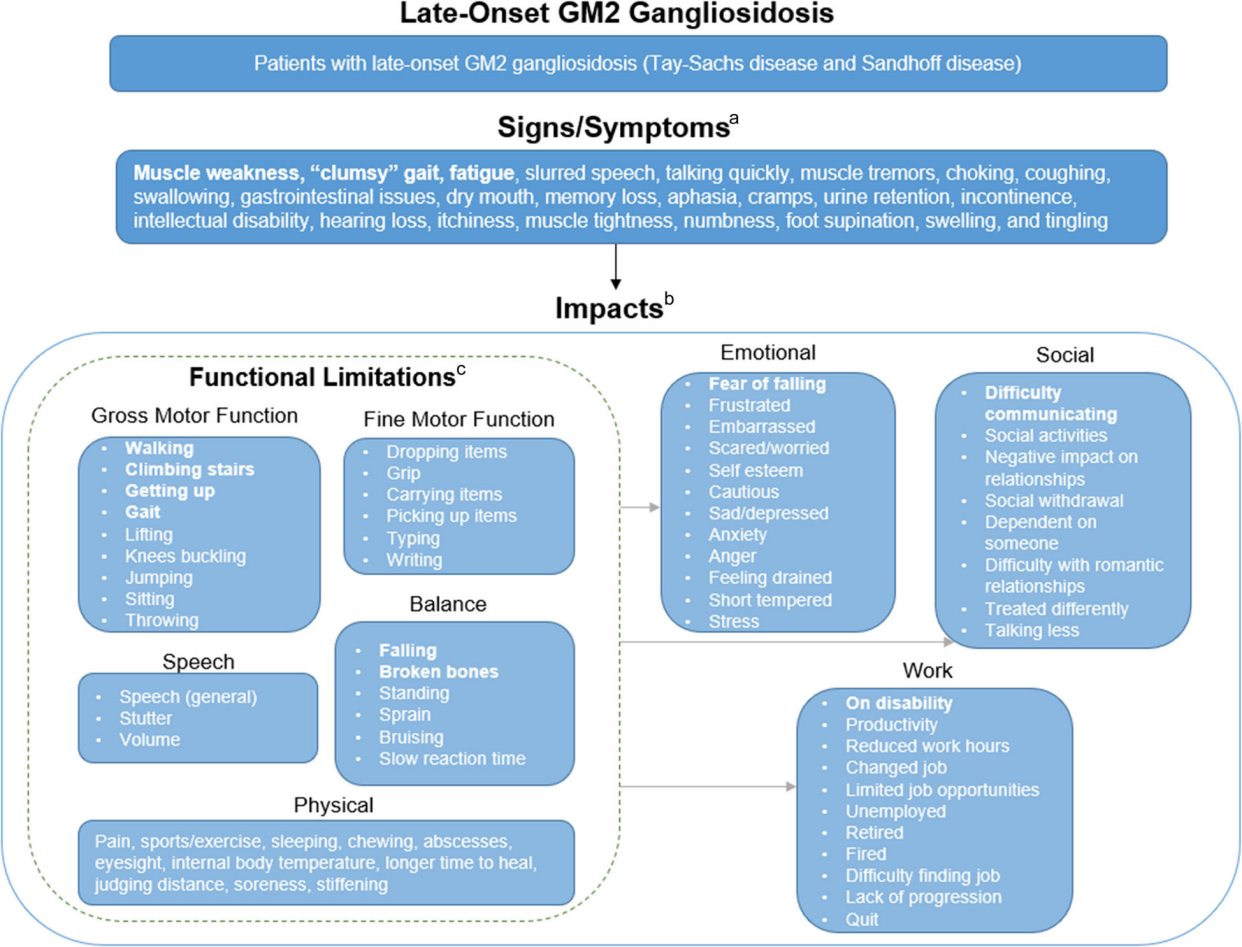
Conceptual Model^a^. ^a^The black arrow indicates a direct relationship between concepts. Grey arrows indicate that emotional, social, and work concepts may also be indirect impacts. ^b^Concepts are in order by frequency of report and concepts reported by ≥ 50% of the sample are bolded. The conceptual model is primarily based on patient interviews using caregiver input as confirmatory. ^c^The dotted box includes all the sub-domains of the functional limitations of late-onset GM2 gangliosidosis

A conceptual model (Fig. 1) was developed using the concepts reported by patients and caregivers in the individual and focus group interviews. An initial conceptual model was developed in 2015 based on the focus group interviews [9]. Following the additional findings from the individual interviews, the conceptual model was redesigned to reflect the changes. All of the concepts listed in the conceptual model were mentioned in interviews, either spontaneously or after probing by the interviewer, and are included in the order of what was most frequently reported. In the model, each arrow indicates the direction of influence. Functional limitations are categorized within the dotted box to include all of its sub-domains: gross motor function, fine motor function, speech, balance, and physical. Due to significant physical limitations, grey arrows lead to emotional, social, and work impacts to indicate that physical limitations also result in these impacts. For example, muscle weakness may affect a patient’s ability to walk, which in turn may result in limited job opportunities. However, the black arrow from symptoms leading to impacts demonstrate that all impacts may be directly affected by symptoms. These findings highlight the most frequently reported symptoms that can be evaluated as potential primary or secondary endpoints in late-onset GM2 gangliosidosis clinical trials and identify the many consequences and impacts of the disease.

## Discussion

This study used a semi-structured interview method that consisted of focus groups and one-on-one interviews to elicit patient perspectives (complemented by caregivers’ views) on the symptoms and functional limitations experienced by patients with late-onset GM2 gangliosidosis. A conceptual model was developed to capture the relationship between symptoms, functional limitations, and other impacts of the disease. As no instruments exist that specifically measure the impact of late-onset GM2 gangliosidosis on the patient experience, this conceptual model may be used to develop new disease-specific clinical outcome assessments (COAs) and inform clinical trial and registry designs.

Patients mentioned experiencing symptoms of muscle weakness and muscle tremors, as well as altered speech (slurred or talking fast), all of which are cited as being more apparent in the first decade of disease manifestation, indicating underlying neurologic problems.^7,8^ In both late-onset Tay-Sachs and Sandhoff diseases, patients described their balance and frequent falling as being “clumsy” (Patient Focus Group #2). Development of a broad-based ataxic gait that made walking difficult is well-documented, and this study captured various adaptations by patients, including assistive devices, specialty adaptations to the home, and changes to gait to assist with mobility.

Some of the symptoms of late-onset GM2 gangliosidosis disclosed by patients and caregivers in this study are similar to those reported elsewhere, particularly problems with balance and walking, muscle weakness, and cognitive function [3, 10]. However, this study sheds light on some of the less common symptoms that patients experience, such as gastrointestinal issues and coughing fits. In addition, it expands upon the knowledge base by utilizing patient descriptions to characterize the impact on patient’s lives and the most bothersome symptoms. The categorization of functional limitations into sub-domains provides evidence on all impacts and its direct and indirect relationship to symptoms of late-onset GM2 gangliosidosis. By separating the symptoms from the functional limitations, the link between symptoms and gross motor, fine motor, balance, speech, and physical impacts is clearly defined and results in a better understanding of the condition. Additionally, our findings indicate that the burden of disease is significant with long-term emotional and social effects that negatively impact patients’ quality of life, highlighting the importance of treatments that effectively address patients’ needs.

The main strength of this study is the patient-centered approach, which focused on generating a representative view from patients and caregivers. Considering the rarity of the disease, a relatively large sample size of 20 patients was attained. The qualitative study design involved both focus group and individual interviews, which minimized any potential limitations that may exist with either method of data collection alone. Data saturation was achieved with the patient interviews, and the caregiver group corroborated the most salient concepts. The consistent input from participants supports the likelihood that the perspectives documented here are representative of patients with late-onset GM2 gangliosidosis. However, it is possible that since the population of this study was drawn from people who attend a disease-specific patient advocacy conference, they may differ from other patients with late-onset GM2 gangliosidosis. Nevertheless, the opportunity to use national patient/family meetings to collect qualitative data from a broad spectrum of patients and caregivers is an important one and represents a patient-centered approach that recognizes patients as the experts of their conditions.

The limitations of this study are those that are common to similar studies in a population of patients with a rare disease, especially one that has limited peer-reviewed literature. The retrospective nature of the research, the self-reporting by parents and other family members, and the presence of disease-related features makes it difficult to exclude recall bias and ascertainment bias. As this study was not designed to evaluate subgroups such as gender or age, there may be different experiences of the disease across populations. Additionally, there were only four patients with late-onset Sandhoff disease who participated in the study, and thus the findings may not fully reflect the experiences of all patients with late-onset Sandhoff disease. However, the four patients with late-onset Sandhoff disease who participated in this study largely reported similar experiences to what was reported by patients with late-onset Tay-Sachs disease. Clinically, late-onset Tay-Sachs and Sandhoff diseases are considered as one entity.

This research has demonstrated that patients experience many consequences of the symptoms of their disease, some of which have a direct impact (e.g., limited mobility and difficulty with speech), while other, more indirect impacts, extend over a longer term (e.g., constant fear of falling or inability to work). The symptoms with the most impact present viable opportunities for evaluation as possible primary or secondary endpoints in late-onset GM2 gangliosidosis clinical trials. Patients were most frustrated by their muscle weakness, lack of independence, altered speech, and lack of/limited mobility, and many expressed a desire to improve these symptoms. In more advanced states of the disease, speech becomes more burdensome for patients as communication becomes more difficult. The conceptual model will be validated in a future study and can provide insight to the medical community. In addition, a repeated measures approach could be useful in order to better understand the trajectory of the condition and how patients are impacted by and cope with it over time.

There is a clear need for additional support and solutions for patients that would enable more independence and management of their own lives. Research into ways to enable patients to move more independently, especially in moderate to advanced disease stages, as well as being able to communicate more effectively, would strongly improve patients’ quality of life by enabling them to be mobile, better understood, and independent.

## Conclusion

This study revealed that patients with late-onset GM2 gangliosidosis experience difficulties with walking, balance, and communication that limit many aspects of their lives, from relationships to career choices. The findings and development of this preliminary conceptual model may prove useful to the recognition or development of instruments that will optimally measure relevant clinical outcomes in clinical trials, registries, or other studies. In the long term, these findings are an important contribution to the current understanding of patients’ and caregivers’ experiences with late-onset GM2 gangliosidosis and may lead to the introduction of interventions designed to better support patients.

## Methods

### Data collection

Sanofi Genzyme collaborated with the National Tay-Sachs and Allied Diseases (NTSAD) patient association in the United States (US) to identify potentially eligible patients and to conduct the interviews. Adult patients with late-onset GM2 gangliosidosis and their caregivers were recruited through their attendance at the NTSAD Annual Family Conference in 2015 and/or 2018. No other screening was conducted to determine eligibility for participation.

### IRB review and informed consent

The protocol, informed consent form, and relevant supporting information were submitted for review and approved by Schulman IRB and Advarra IRB. The study was conducted in accordance with the ethical principles outlined in the Declaration of Helsinki and consistent with Good Clinical Practice and applicable regulatory requirements.

Informed consent was obtained in writing from interested patients and caregivers prior to participation in the focus groups and individual interviews. To preserve patient confidentiality, all transcripts and audio recordings were de-identified and no personal identifying information was collected.

### Interviews

A qualitative research design using semi-structured focus group and individual interviews with patients and caregivers was adopted to elicit multiple perspectives on each topic [11]. In 2015, two patient focus groups and one caregiver focus group were conducted with study participants. A literature review of the signs, symptoms, and impacts of these diseases was used as the basis for the development of a discussion guide. The discussion guide was used as an aid to stimulate a free-flowing discussion aimed at eliciting the most frequently occurring and bothersome signs and symptoms, as well as the most salient consequences of late-onset GM2 gangliosidosis. Prior to participation, patients and caregivers enrolled in the study completed a de-identified questionnaire relating to demographic data and clinical history.

Each focus group comprised between five and seven participants. To ensure homogeneity between focus group participants, the GM2 gangliosidosis patient groups differed by age distribution, time since diagnosis, and living situation: one group with older and more advanced patients (Patient Focus Group #1), and the other one (Patient Focus Group #2) with primarily younger patients diagnosed within the previous 5 years and living alone or with parents. The sessions were audio recorded with participant permission to capture the discussion verbatim in the transcripts in order to be analyzed a posteriori. The patient focus groups were designed to improve the understanding of the signs, symptoms, and burden of disease by obtaining the views directly from the perspective of patients with late-onset GM2 gangliosidosis. To further confirm the symptoms and impacts that were experienced by patients, a single focus group interview was held with seven caregivers of some of these patients. This focus group was designed to expand upon the concepts spontaneously elicited by patients and obtain information on concepts that were not fully discussed with patients.

In 2018, 18 interviews were conducted with patients with late-onset GM2 gangliosidosis, nine of whom had also participated in the 2015 focus group interviews. All of the interviews were conducted one-on-one, with the exception of five patients who were interviewed with their caregivers assisting in communicating the full breadth of their experience. The interviews lasted approximately 75 min and the goal was to confirm the most salient symptoms and impacts for patients with GM2 gangliosidosis.

### Data analysis and statistics

Data were pooled across the 2015 and 2018 interview cohorts to describe the symptoms and functional limitations of GM2 gangliosidosis. While this included data from the nine patients who participated in both the focus group and individual interviews, their 2018 data was retained in the analysis because the one-on-one interviews provided a more in-depth assessment of their experiences. There also was the possibility that patient experiences may have changed in the three-year period between the interviews. The initial analytic step involved repeatedly listening to recordings and reading transcripts while taking notes, and comparing the recordings and transcripts with the minutes made during the focus group sessions. Meaningful information was organized into themes that described and categorized the possible observations. De-identified interview transcripts were reviewed and analyzed in ATLAS.ti 8 software using content analysis techniques to extract meaningful themes and concepts. The frequency with which themes and concepts were mentioned was the primary method for establishing the salience of their relevance to GM2 patients. The transcript from each focus group was treated as a single patient in analysis, as it was not possible to distinguish the identities of individual speakers from the focus group transcripts.

Concept saturation was assessed to ensure that the number of interviews was sufficient to confirm that all of the most relevant concepts related to the late-onset GM2 gangliosidosis patient experience were identified.

## Data Availability

Data generated from this study are available from the corresponding author and with permission of Sanofi Genzyme on reasonable request.
